# The effect of parental marital status on non-suicidal self-injurious behavior among middle school students in rural western China: a multicenter cross-sectional study based on propensity score matching method

**DOI:** 10.3389/fpsyt.2025.1644937

**Published:** 2025-10-07

**Authors:** Xiaohong Ren, Lu Pan, Yuqin Song, Ping He, Dapeng Wu, Jiaming Luo

**Affiliations:** ^1^ Department of Hematology, The Affiliated Hospital of North Sichuan Medical College, Nanchong, Sichuan, China; ^2^ School of Psychiatry, North Sichuan Medical College, Nanchong, Sichuan, China

**Keywords:** parental divorce, non-suicidal self-injury, mental health, middle school students, propensity score

## Abstract

**Introduction:**

Parental marital status is a pivotal determinant of adolescent maladaptive behaviors. However, the specific correlation between parental marital status and Non-Suicidal Self-Injury (NSSI) among adolescents in western China has not been thoroughly investigated.

**Methods:**

A cross-sectional study was conducted involving 7,274 students from non-divorced families and 1,332 from divorced families. Participants were assessed using the Ottawa Self-Injury Inventory (OSI) and the Depression, Anxiety, and Stress Scale-21 Items (DASS-21). Propensity score matching (PSM) was applied to control for confounders, and logistic regression was used to analyze associations.

**Results:**

The prevalence of non-suicidal self-injury (NSSI) was higher in adolescents from non-divorced families (4.1%) than in those from divorced families (1.6%). However, propensity score matching analysis revealed that parental divorce was associated with a significantly increased odds of NSSI after controlling for confounders. Furthermore, depression and anxiety were identified as independent risk factors for NSSI. Mediation analysis indicated that the emotional distress (depression, anxiety, and stress) did not significantly mediate the relationship between parental divorce and NSSI. Additional significant predictors of NSSI included girls and age.

**Discussion:**

Adolescents from divorced families in western China show a higher incidence of NSSI, though this relationship is not mediated by emotional distress. These findings highlight the need for families and society to enhance attention to the psychological well-being of this vulnerable adolescent population.

## Introduction

NSSI is defined as the intentional and socially unacceptable infliction of harm to one’s own body without the intent to commit suicide (1). Numerous studies have demonstrated that engagement in NSSI significantly increases the risk of suicidal ideation, suicide attempts, and a range of mental disorders (2, 3). The detrimental consequences of NSSI extend beyond the individual, profoundly impacting familial dynamics, interpersonal relationships, and in extreme cases, resulting in fatal outcomes (4). Adolescents are particularly susceptible to NSSI (5), rendering self-injury a critical global public health issue that demands immediate attention and intervention.

Globally,a previous meta-analysis indicated that globally, 8.3% of adolescents engage in occasional non-suicidal self-injury, while 20.3% exhibit repeated non-suicidal self-injury (6). In China, a meta-analysis of middle school populations reveals an aggregate NSSI prevalence of 22.4% (7), though marked regional disparities are evident. Provincial-level studies illustrate this heterogeneity: the incidence among rural primary and secondary school students in Jiangxi Province is 14.8% (8), while the lifetime prevalence of NSSI among adolescents in Yunnan Province is 47.1% (9). Notably, despite these documented variations, the epidemiological profile of NSSI in Western China’s adolescent population remains poorly characterized.

Previous studies have identified a range of factors associated with NSSI, including personal and family factors, social environment, and psychological conditions (10, 11), all of which significantly affect adolescents’ physical and psychological well-being. Research has revealed a strong link between NSSI and depression, with adolescents exhibiting depressive symptoms showing significantly higher vulnerability to NSSI compared to their non-depressed counterparts (12, 13). Furthermore, anxiety has been identified as a crucial mediating factor in the relationship between adverse life events and NSSI (14), and elevated anxiety levels often act as significant triggers for NSSI episodes (15, 16). When individuals encounter stressful life events, the likelihood of engaging in NSSI increases, particularly if they are in a negative mental health state, as NSSI can serve as a coping strategy to alleviate the negative emotions stemming from these events (17). Beyond these established factors, researchers have also explored other potential risk factors for NSSI, such as borderline personality disorder, externalizing symptoms (e.g., aggression and behavioral problems), impulsivity, self-prediction (i.e., self-reported likelihood of engaging in NSSI in the future), and gender. However, studies have reported significant variations in these factors, and intervention programs targeting known risk factors often yield only moderate results. These limitations underscore the necessity for further research to identify novel risk factors and develop more targeted prevention strategies, thereby advancing our understanding and improving clinical outcomes in NSSI management.

Divorce has emerged as a widespread and increasingly prevalent social phenomenon, with substantial variations in divorce rates observed across different countries and regions (18). In China, the divorce rate has experienced a rapid escalation in recent years, a trend that can be attributed to evolving societal attitudes, enhanced educational attainment among women, and improvements in female economic independence (19, 20). Research has consistently demonstrated that parental separation or divorce constitutes a significant risk factor for children’s psychological well-being, predisposing them to various mental health challenges (21).These include increased susceptibility to depression (21, 22), elevated risk of suicidal ideation (21), and greater vulnerability to suicide attempts. Such psychological distress may subsequently contribute to a higher propensity for NSSI. This pattern is particularly evident in South Korea, where adolescents from divorced or remarried families demonstrate significantly elevated risks of engaging in NSSI behaviors (23, 24). In Lebanon, adolescents from separated families demonstrate significantly higher levels of social anxiety, avoidance behaviors, depression, and suicidal ideation (25).A Taiwanese study further emphasized that dysfunctional family dynamics substantially increase the risk of NSSI among high school students (26). Similarly, research conducted in Hong Kong has established parental marital discord as a critical risk factor for the chronic persistence of emotional and behavioral issues in adolescents (27). These findings collectively underscore the significant influence of parental marital status on adolescent NSSI behaviors. Middle school students, who are navigating a critical phase of emotional and psychological development, are particularly vulnerable to external stressors such as academic pressure and emotional instability. During this sensitive period, the marital status of their parents can exert an even more pronounced impact on their mental health, especially in relation to the development and maintenance of NSSI behaviors.

The western region of China faces slower economic and social development compared to other areas, coupled with a substantial population of children and adolescents. However, research on NSSI among adolescents in Sichuan remains limited, particularly studies investigating the influence of parental marital status on adolescent NSSI behaviors. To address this gap, and after controlling for potential confounding variables using Propensity Score Matching (PSM), we propose the following two hypotheses regarding NSSI among adolescents in rural western China: Hypothesis 1: Adolescents from divorced families show a significantly higher rate of non-suicidal self-injury compared to those from intact families. Hypothesis 2: Emotional distress (including depression, anxiety, and stress) mediates the association between parental divorce and non-suicidal self-injury among adolescents.

## Methods

### Survey and inclusion criteria

This study employs a cross-sectional design to investigate the prevalence and characteristics of NSSI among adolescents. The statistical analysis utilizes a two-tailed test with a significance level (*α)* of 0.05 and an allowable margin of error (*δ*) of 0.02. Based on the most recent epidemiological data from China, which reports an adolescent NSSI prevalence rate of 22.4% (7). We calculated the minimum required sample size via PASS15 software. The initial calculation yielded a minimum sample size of 6,678 participants. To account for potential data attrition (estimated at 20%) due to incomplete responses, participant withdrawal, and other methodological factors, we adjusted the target sample size to 8,343 participants.


$$n=\frac{Z^{2}\cdot p\cdot (1-p)}{\delta^{2}}$$

The study data were collected during March and April 2020 through a comprehensive survey of middle school students across 20 towns in the target region. To ensure representativeness, the study design incorporated stratification based on key demographic variables, including population size, economic development levels, and educational resource distribution. A stratified random sampling approach was implemented, with schools serving as the primary sampling unit. Specifically, one school was randomly selected from each of the 17 smaller towns, while two schools were chosen from each of the three larger towns, yielding a total of 23 participating institutions.

The research team utilized the Wenjuanxing (WJX) platform (website: https://www.wjx.cn) for data collection. After uploading the questionnaire, a unique QR code was generated for survey access. School administrators distributed this QR code to parents or legal guardians through established communication channels. Participants were required to provide informed consent before participation, with the consent form displayed as the initial page of the digital survey interface. Only after electronically signing the consent form could participants proceed to complete the questionnaire. To minimize reporting bias, an anonymous online survey was conducted. Participants were assured of the strict confidentiality of their data and were informed that the information would be used solely for scientific research purposes. This study was in accordance with the ethical principles, reviewed by the Ethics Committee of Nanchong Psychosomatic Hospital.

### Data collection and measurement

The data were primarily collected from middle schools in Yingshan County, a remote region in western China. This location was selected due to its representativeness of rural areas in western China, where the majority of families live in poverty, engage in agricultural labor, and reside in either villages or nearby towns.

Data collection was conducted in two stages. In the first stage, researchers underwent comprehensive training to familiarize themselves with the study procedures, questionnaire content, and form completion protocols. They also collaborated with class teachers of the participating classes to discuss the research objectives and questionnaire content, aiming to improve response rates and ensure data quality. Following unanimous consent from all stakeholders, the questionnaires were distributed. In the second stage, two researchers collected the completed questionnaires and conducted a preliminary review to verify data accuracy. The results were exported from the WJX platform into an Excel spreadsheet for further processing. Prior to analysis, missing values, obvious errors, and inconsistent responses were systematically removed, retaining only data that met the predefined inclusion criteria for analysis.

The research team comprised practicing physicians from the Departments of Psychiatry and Psychology of Affiliated Hospital of North Sichuan Medical College, as well as graduate students from the psychiatry program at the same institution. Before initiating the survey, all team members received systematic training to ensure familiarity with the procedures, content, and subsequent steps. Throughout the study, researchers strictly adhered to Standard Operating Procedures (SOP) to maintain consistency and reliability.

The study collected 8,785 questionnaires, retaining 8,606 (98.0% validity rate) after excluding 176 based on predefined criteria: lack of consent, missing data, or inconsistent responses. Analysis focused on adolescents aged 12-18 (inclusive of 12 and 18 years old) from divorced families, with inclusion requiring: (1) age 12-18, (2) parental divorce/separation, and (3) ≥1 year since separation to ensure stable family structure and measurable psychological impact.

### Demographic characteristics

A self-designed questionnaire was utilized to collect socio-demographic information from middle school students. Including age, gender, only-child status, home location (rural or urban), boarding school(in China, middle schools provide dormitories, and students may choose to board based on their family circumstances), left-behind status (defined as individuals under 18 years old who reside in their household registration location for six months or longer due to one or both parents working away and being unable to live with both parents), education level ((junior high school, senior high school)), father’s occupation, paternal education, maternal education, mother’s occupation, and parental marital status (individuals not living with both parents due to separation or divorce were categorized as having experienced parental divorce).

### NSSI assessment tool

The Ottawa Self-Injury Inventory (OSI) (28), originally developed by Nxion and Paula Cloutier, is a comprehensive psychometric instrument designed to assess multiple dimensions of NSSI, including behavioral characteristics, underlying motivations, frequency patterns, and addictive components. Extensive international validation studies have established its reliability and validity as an effective assessment tool for NSSI (28). For the current study, we employed the Chinese version of the Ottawa Self-Injury Inventory (OSI Chinese version), which has demonstrated robust psychometric properties in previous research. Specifically, the Chinese adaptation has shown excellent test-retest reliability (coefficient > 0.400) and strong construct validity, with subscale internal consistency reaching a Cronbach’s *α* coefficient of 0.952 (29). In our sample, the questionnaire demonstrated high reliability, with a Cronbach’s α coefficient of 0.864. NSSI identification was operationalized according to the DSM-5 diagnostic criteria for NSSI, supplemented by relevant items from the OSI inventory. Participants were classified as exhibiting NSSI behaviors if they reported engaging in one or more of the following behaviors (e.g., cutting, burning, scratching, hitting, biting) within the preceding 12 months.

### Mental health issues

The Depression, Anxiety, and Stress Scale (DASS-21) was employed to evaluate negative emotional states among left-behind children. The Likert scale scores range from 0 to 3 ((0 = never to 3 = almost always), This validated instrument comprises 21 items across three subscales: depression (items 3, 5, 10, 13, 16, 17, and 21), anxiety (items 2, 4, 7, 9, 15, 19, and 20), and stress (items 1, 6, 8, 11, 12, 14, and 18). The maximum and minimum scores for each dimension are 21 and 0, respectively, and they are positively correlated with the corresponding emotional levels. The internal consistency of this scale, measured by Cronbach’s alpha, was 0.956, with the subscale coefficients exceeding 0.800 (30). The Chinese version of the scale effectively reflects the emotional states of residents, university students, and adolescents in mainland China (31, 32), In this study, the alpha coefficients for the anxiety, depression, and stress dimensions of the DASS-21 were all greater than 0.830, further supporting its cross-cultural validity (33).making it a valuable instrument for assessing negative emotional states.

### Statistical analyses

#### Propensity score matching

In this study, significant differences in demographic characteristics were observed between students from divorced and non-divorced parental groups. To address these differences and mitigate potential confounding effects on the study outcomes, we employed Propensity Score Matching (PSM) to balance the baseline characteristics between the two groups (34). PSM is a robust statistical method that minimizes discrepancies in demographic characteristics between the experimental and control groups, thereby reducing bias introduced by confounding variables (35).Specifically, the PSM procedure was implemented in three steps. First, a set of observed covariates (i.e., confounders) was identified based on theoretical and empirical considerations. Second, a Logit model was used to estimate the probability of participants being assigned to the divorced parental group, known as the propensity score. Finally, a matching algorithm was applied to pair each participant in the divorced parental group with a participant in the non-divorced parental group who had a similar propensity score. This matching process ensured that the two groups were balanced with respect to potential confounding factors, enhancing the validity of the comparative analysis.

**Figure 1:**
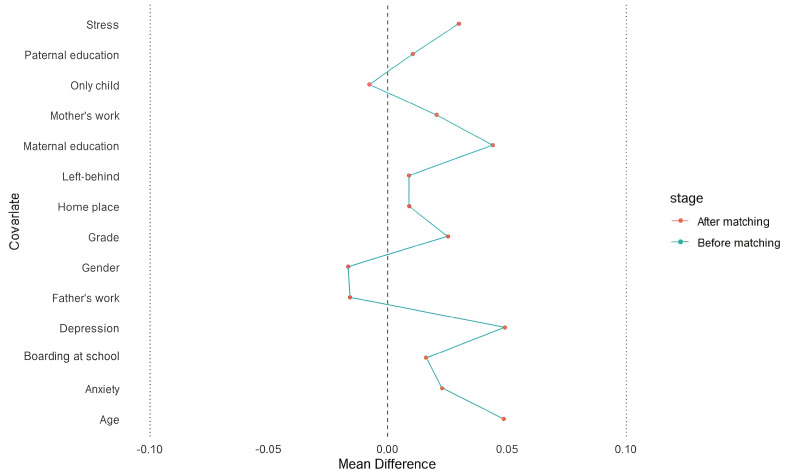
Description of the SMD for each potential covariate before and after matching.

**Figure 2:**
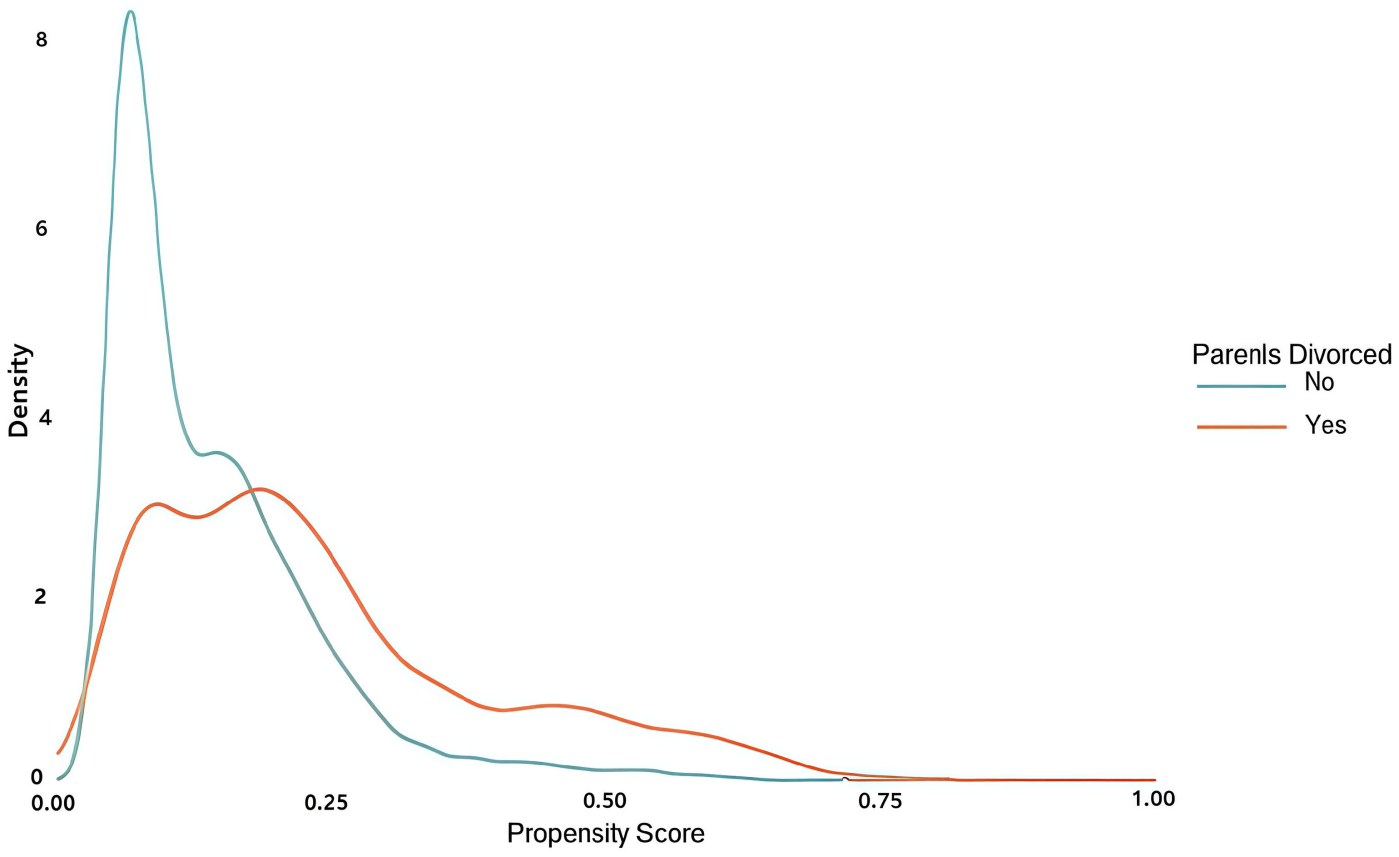
Distribution of propensity scores before matching.

**Figure 3:**
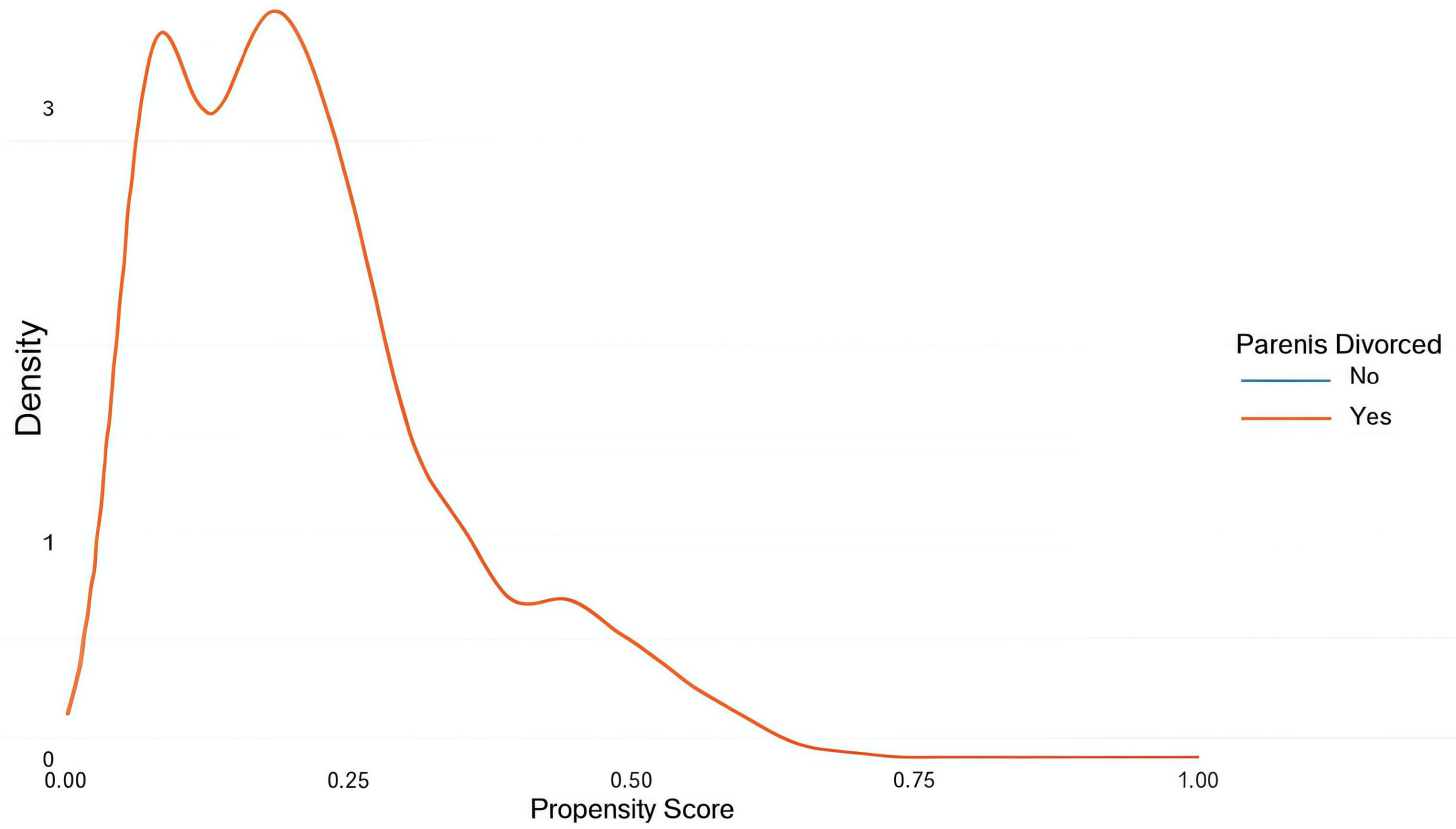
Distribution of propensity scores after matching.

PSM was performed using R 4.2.2 with the MatchIt, tableone, and cobalt packages. The Logit model was selected for the matching function, with a caliper set at 0.2*SD (36, 37). A greedy nearest neighbor matching approach without replacement was used for 1:1 matching. The standardized mean difference (SMD) was employed to assess the balance of demographic characteristics after matching (34, 38). To achieve a satisfactory matching balance, the absolute value of the SMD was set to be less than 0.1 (36, 38, 39). The balance of confounding factors before and after matching is illustrated using an SMD plot (**Figure 1**), while the distribution of propensity scores before and after matching for both the divorced and non-divorced parental groups is shown in **Figures 2**, **3**.

### Data analysis

Univariate analyses were conducted using SPSS 26.0 both before and after matching. The Kolmogorov-Smirnov test (K-S test) revealed that the data were not normally distributed. Consequently, non-parametric tests were utilized for between-group comparisons to evaluate median differences, the Wilcoxon rank-sum test was applied to compare non-normally distributed continuous and ordinal variables across groups. Additionally, binary logistic regression analysis was performed to identify potential risk factors, with model adequacy verified through the Hosmer-Lemeshow test (*P* > 0.05 indicating satisfactory fit), Mediation analyses were conducted using SPSS PROCESS Macro (v4.1). All statistical tests were two-tailed, with a significance level set at *α* = 0.05.

## Results

### Analysis of NSSI

In this study, 7,274 middle school students with non-divorced parents and 1,332 middle school students with divorced parents participated in the survey and completed the assessment. The prevalence of NSSI was found to be 4.1% in the non-divorced parental group, and 1.6% in the divorced parental group. There were statistically significant differences between the two groups (divorced vs. non-divorced parents) in terms of age, grade, left-behind experience, Boarding at school, only child status, residence place, Paternal educational qualification, Maternal educational qualification, father’s occupation, mother’s occupation (*P<*0.01, **Table 1**).

**Table 1:** General situation analysis of NSSI behavior among middle school students.

Variable names	Level	Overall	Non – NSSI (N = 8120)	NSSI (N = 486)	χ²/Z	*P*
Grade	Junior high	5937 (69.0)	5658(65.7)	279(3.2)	31.709	<0.001
	Senior high	2669 (31.0)	2462(28.6)	207(2.4)		
Gender	Boys	4540 (52.8)	4379(50.9)	161(1.9)	78.772	<0.001
	Girls	4066 (47.2)	3741(43.5)	325(3.8)		
Divorced Parents	No	7274(84.5)	6922(80.4)	352(4.1)	57.596	<0.001
	Yes	1332(15.5)	1198(13.9)	134(1.6)		
Only child	No	7400 (86.0)	6986(81.2)	414(4.8)	0.209	0.648
	Yes	1206 (14.0)	1134(13.2)	72(0.8)		
Residence place	Urban	4660 (54.1)	4418(51.3)	242(2.8)	3.933	0.053
	Rural	3946 (45.9)	3702(43)	244(2.8)		
Boarding at school	No	5902 (68.6)	5603(65.1)	299(3.5)	11.562	0.001
	Yes	2704 (31.4)	2517(29.2)	187(2.2)		
Left-behind children	No	5074 (59.0)	4812(55.9)	262(3.0)	5.209	0.022
	Yes	3532 (41.0)	3308(38.4)	224(2.6)		
Fathers’ occupation	Farmers	757 (8.8)	1555(18.1)	40(0.5)	1.343	0.252
	Workers	1648 (19.1)	717(8.3)	107(1.2)		
	Merchant	4608 (53.5)	713(8.3)	261(3.0)		
	Civil servant	1593 (18.5)	1515(17.6)	78(0.9)		
Paternal educational qualification	Primary education or below	1796 (20.9)	1685(19.6)	111(1.3)	3.226	0.192
	Secondary education	6464 (75.1)	6102(70.9)	362(4.2)		
	Associate’s degree or equivalent	346 (4.0)	333(3.9)	13(0.2)		
Maternal educational qualification	Primary education or below	2915 (33.9)	2721(31.6)	194(2.3)	0.756	0.008
	Secondary education	5461 (63.5)	5177(60.2)	284(3.3)		
	Associate’s degree or equivalent	230 (2.7)	222(2.6)	8(0.1)		
Mother’s occupation	Farmers	617 (7.2)	580(6.7)	37(0.4)	0.838	0.078
	Workers	1894 (22.0)	1766(20.5)	128(1.5)		
	Merchant	4631 (53.8)	4381(50.9)	250(2.9)		
	Civil servant	1464 (17.0)	1393(16.2)	71(0.8)		
Age		14(12,18)	14(12,18)	15(12,18)	-5.320	<0.001
Depression		2(0,21)	1(0,21)	7(0,21)	-23.488	<0.001
Anxiety		1(0,21)	1(0,21)	6(0,21)	-22.844	<0.001
Stress		2(0,21)	2(0,21)	7(0,21)	-21.232	<0.001

### Potential confounder selection for propensity score matching

Based on the relationship between the grouping variables and outcome variables, potential confounding factors such as grade, left-behind experience, residence place, Paternal educational qualification, Maternal educational qualification, and age were selected as covariates and matched in the propensity score model.

### Propensity score matching

The PSM procedure resulted in a final matched sample of 2,664 students, comprising 1,332 from divorced parental families and 1,332 from non-divorced parental families. After PSM, the incidence rate of NSSI was 3.4% in the non-divorced parental group and 5.0% in the divorced parental group. The standard deviation of the propensity scores for both groups was 0.0001, indicating a high level of balance achieved through the matching process.

Potential confounding factors, such as grade, left-behind experience, residence place, Paternal educational qualification, Maternal educational qualification, age, and the variables not included as potential confounders, demonstrated excellent balance (all SMD < 0.1; see **Table 2**). This suggests that the matching process effectively balanced all potential confounders between the two groups.

**Table 2:** Demographic characteristics of the whole sample by parenta’l marriage.

Variables		Before PSM		*χ²/Z*	*P*	After PSM		*x^2^/Z*	*P*
		Non-divorced parents (N = 7274)	Divorced parents (N = 1332)			Non-divorced parents (N = 1332)	Divorced parents (N = 1332)		
Gender	Boys	3855 (53.0)	685 (51.4)	1.052	0.305	703 (52.8)	685 (51.4)	10.943	<0.001
	Girls	3419 (47.0)	647 (48.6)			629 (47.2)	647 (48.6)		
Grade	Junior high	5159 (70.9)	778 (58.4)	81.840	<0.001	775 (58.2)	778 (58.4)	4.579	0.937
	Senior high	2115 (29.1)	554 (41.6)			557 (41.8)	554 (41.6)		
Left-behind	yes	2071(37.1)	831(62.5)	295.718	<0.001	565 (42.4)	831 (62.4)	0.006	<0.001
Boarding at school	yes	2123 (29.2)	581 (43.6)	108.162	<0.001	597 (44.8)	581 (43.6)	1.777	0.558
Only child	yes	815 (11.2)	391 (29.4)	306.286	<0.001	386 (29.0)	391 (29.4)	0.029	0.865
Residence place	City	4107 (56.5)	553 (41.5)	100.677	<0.001	551 (41.4)	553 (41.5)	3.097	0.969
	Rural	3167 (43.5)	779 (58.5)			781 (58.6)	779 (58.5)		
Paternal educational qualification	Elementary school	1469 (20.2)	327 (24.5)	16.897	<0.001	311 (23.3)	327 (24.5)	1.419	0.661
	Secondary school	5497 (75.6)	967 (72.6)			987 (74.1)	967 (72.6)		
	College or above	308 (4.2)	38 (2.9)			34 (2.6)	38 (2.9)		
Maternal educational qualification	Elementary school	2362 (32.5)	553 (41.5)	52.950	<0.001	559 (42.0)	553 (41.5)	1.558	0.174
	Secondary school	4695 (64.5)	766 (57.5)			749 (56.2)	766 (57.5)		
	College or above	217 (3.0)	13 (1.0)			24 (1.8)	13 (1.0)		
Father’s occupation	Farmer	679 (9.3)	78 (5.9)	29.624	<0.001	97 (7.3)	78 (5.9)	7.768	0.158
	Worker	1397 (19.2)	251 (18.8)			247 (18.5)	251 (18.8)		
	Businessman	3820 (52.5)	788 (59.2)			744 (55.9)	788 (59.2)		
	Public servant	1378 (18.9)	215 (16.1)			244 (18.3)	215 (16.1)		
Mother’s occupation	Farmer	556 (7.6)	61 (4.6)	39.625	<0.001	97 (7.3)	61 (4.6)	12.334	0.006
	Worker	1577 (21.7)	317 (23.8)			304 (22.8)	317 (23.8)		
	Businessman	3849 (52.9)	782 (58.7)			725 (54.4)	782 (58.7)		
	Public servant	1292 (17.8)	172 (12.9)			206 (15.5)	172 (12.9)		
NSSI	YES	352(4.1)	134(1.6)	56.618	<0.001	100(3.8)	134(5.0)	21.848	<0.001
Age		14(12,18)	15(12-18)	-6.181	<0.001	15(12-18)	15(12-18)	-0.106	0.309
Depression		2(0,21)	4(0,21)	-8.755	<0.001	4(0,21)	4(0,21)	-6.027	<0.001
Anxiety		2(0,21)	4(0,21)	-8.575	<0.001	2(0,21)	4(0,21)	-5.728	<0.001
Stress		4(0,21)	6(0,21)	-7.468	<0.001	4(0,21)	6(0,21)	-5.136	<0.001

The SMD values for each potential confounding factor before and after matching are illustrated in **Figure 1**. After matching, all confounders exhibited excellent balance (all SMD < 0.1). Additionally, the SMD values for all confounders and their subgroups indicated strong matching both before and after PSM (all SMD < 0.1). Furthermore, the symmetry of propensity scores between the divorced and non-divorced groups improved significantly after matching, as visually demonstrated in **Figures 2**, **3**.

### Demographic and clinical characteristics of the matched sample

**Table 2** shows the demographic and clinical characteristics of the matched samples after propensity score matching. Univariate analysis indicates that after matching, the divorced and non-divorced groups are well-balanced in grade, left-behind experience, residence place, Paternal educational qualification, Maternal educational qualification, and age (*P* > 0.05, all SMD < 0.1). Regarding the DASS-21 dimensions, namely depression (Z = 39.283, *P* = 0.011), anxiety (Z = 42.824, *P* = 0.048), and stress (Z = 30.797, *P* = 0.013), there were statistically significant differences between the divorced and non-divorced groups. Specifically, the divorced group exhibited higher scores in depression, anxiety, and stress compared to the non-divorced group.

### Multivariable analyses

We employed binary logistic regression to examine the relationship between parental marital status and NSSI, adjusting for depression, anxiety, and stress as covariates. The results of the analysis revealed that several factors were significantly associated with NSSI among middle school students. Parental divorce (OR = 4.052, 95% CI: 2.629~6.245, P < 0.001), depression (OR = 1.061, 95% CI: 1.029~1.095, P < 0.001), anxiety (OR = 1.074, 95% CI: 1.038~1.111, P < 0.001), girls (OR = 2.463, 95% CI: 1.704~3.559, P < 0.001), and age (OR = 1.127, 95% CI: 1.006~1.262, P = 0.039) were all significantly linked to an increased likelihood of engaging in NSSI (**Table 3**). The Hosmer-Lemeshow test (*P*>0.05) confirmed that the model had a good fit.

**Table 3:** Binary logistic regression of NSSI in middle school students.

Variable	β	SE	Wald χ2	*P* value	OR	95% CI
(Intercept)	-7.158	0.919	60.595	<0.001	0.001	
Divorced Parents	1.399	0.221	40.195	<0.001	4.052	2.629~6.245
Depression	0.06	0.016	14.021	<0.001	1.061	1.029~1.095
Anxiety	0.071	0.017	17.263	<0.001	1.074	1.038~1.111
Girls	0.901	0.188	23.000	<0.001	2.463	1.704~3.559
Age	0.120	0.058	4.271	0.039	1.127	1.006~1.262

### Mediation analyses

Using the SPSS PROCESS Macro (v4.1), separate mediation models were constructed to examine the potential mediating roles of depression, anxiety, and stress. Parental divorce (YES vs. No) was included as the independent variable, and NSSI (YES vs. No) as the dependent variable, with depression, anxiety, and stress tested as individual mediators. All analyses were conducted using a propensity score-matched sample to ensure comparability between groups.

The results indicated that parental marital status did not significantly predict levels of depression (β = 0.173, *P* = 0.584), anxiety (β = 0.234, *P* = 0.423), or stress (β = 0.302, *P* = 0.348). However, depression (β = 0.108, *P* < 0.001), anxiety (β = 0.112, *P* < 0.001), and stress (β = 0.101, *P* < 0.001) each significantly positively predicted NSSI. After controlling for these emotional variables, the direct effect of parental marital status on NSSI remained significant (all *P* < 0.05). Furthermore, all indirect effect Bootstrap confidence intervals included zero, indicating that depression, anxiety, and stress did not show significant mediating roles in the relationship between parental marital status and NSSI.

## Discussion

This study employs PSM methodology to examine the relationship between parental marital status and NSSI among Chinese adolescents in western regions, effectively addressing selection bias concerns that have limited previous observational research. Through rigorous analysis of matched samples, we found parental divorce is independently associated with NSSI risk, even after controlling for key psychological covariates. Initial comparisons in unmatched samples revealed statistically significant disparities (*P* < 0.05) between adolescents from divorced and non-divorced families across multiple mental health dimensions, including NSSI frequency, depressive symptoms, anxiety levels, and stress. Following rigorous PSM adjustment for potential confounders, robust between-group differences persisted, with adolescents from divorced families demonstrating a 4-fold increased risk of NSSI engagement compared to their counterparts from intact families. Interestingly, although a significant association was observed between parental divorce and increased risk of NSSI, unlike previous studies (40), the present study did not find that this relationship was mediated by elevated levels of emotional distress in adolescents. This suggests that the link between parental divorce and NSSI is more likely attributable to direct pathways or moderated by other contextual factors. Future research should further explore these potential moderating mechanisms to more comprehensively understand how family structure risks translate into individual behavioral issues. These methodologically rigorous findings not only address a critical gap in regional mental health research but also advance our understanding of family system dynamics in adolescent NSSI within China’s unique sociocultural context.

Compared to students from non-divorced families, those from divorced families exhibit a higher prevalence of NSSI behaviors (41). Divorce is the outcome of a separation process that often begins even before parents leave the household, with long-lasting effects (42). The quality of the emotional bond between parents and children plays a significant role in the onset of adolescent NSSI (43).According to Family Process Theory, factors such as post-divorce family conflict (44),chaotic parenting practices, and diminished parental monitoring (45), exert a more critical influence on adolescents’ psychological adjustment than divorce itself. After divorce, decreased parental supervision may increase the risk of NSSI (46). Middle school students from divorced families are more likely to experience both internalizing and externalizing behavioral problems than their peers from non-divorced families. Additionally, societal attitudes toward divorce remain largely negative, and adolescents from divorced families may experience higher levels of distress (21). The divorce process typically exposes both parents and children to distinct stressors at different stages, often resulting in emotional detachment and progressive marital dissatisfaction (42). This familial disruption can lead to impaired family functioning and adverse psychological consequences, potentially compromising children’s mental health outcomes (47). From an economic perspective (48), divorce often leads to reduced household financial resources, and the resulting financial strain may further contribute to risk behaviors among adolescents. Evidence suggests that adolescents from divorced families are particularly vulnerable to various psychological difficulties, including heightened anxiety (22), depression (40), aggression, stress, behavioral disturbances, substance abuse, and suicidal ideation (22). Meanwhile, social stigma and support mechanisms warrant equal attention: adolescents may experience a sense of shame associated with changes in family structure, and the breakdown of social support networks can amplify psychological distress and engagement in NSSI (41).These findings highlight the significant impact of parental marital status on NSSI behaviors among middle school students.

Our findings substantiate a significant association between NSSI and depressive symptoms, which aligns with findings from previous research (49, 50). According to the emotion regulation disorder theory, individuals who lack effective strategies for regulating negative emotions are more likely to engage in NSSI behaviors as a way to alleviate emotional pain and distress (51). Over time, persistent difficulties in emotion regulation can lead to the development of depressive symptoms, creating a vicious cycle. Klonsky (52) found that individuals who engage in NSSI typically experience higher levels of depressive symptoms, and there is a strong, bidirectional relationship between the two, with mutual reinforcement. Family environmental factors also play a crucial role in this process. Research suggests that family dysfunction can make individuals more vulnerable to emotion regulation difficulties, which in turn increases the risk of both NSSI behaviors and depressive symptoms (53). Living in a single-parent household often results in limited economic and social resources (such as support and parental supervision), which can be linked to poorer mental health outcomes in children, including depression (54) and NSSI (55). Previous studies have shown that 40.4% of adolescents from separated families in Sweden suffer from long-term depression (54), while a study in Galicia, Spain, revealed that parental separation leads to an average increase of 20% in depressive symptoms, generalized anxiety, hostility, paranoid thoughts, and interpersonal alienation (56).

Our findings corroborate previous research demonstrating a strong association between anxiety symptoms and NSSI in adolescents (22). From a cognitive-affective perspective, adolescence represents a critical developmental period characterized by rapid physical and emotional changes, during which self-identity and emotion regulation capacities are still evolving. When confronted with stressors or negative emotions, adolescents often lack effective coping mechanisms, leading to heightened anxiety (57). This emotional dysregulation, coupled with low self-directedness in managing challenges, may manifest in self-harming behaviors (55). Adolescents from divorced families appear particularly vulnerable to anxiety-related difficulties. Research conducted in Lebanon revealed that parental separation is associated with elevated social anxiety and generalized anxiety symptoms, often stemming from feelings of insecurity and environmental instability (25). These anxiety-provoking experiences may contribute to social avoidance behaviors and, ultimately, increase the risk of NSSI episodes (15, 16). Consistent with prior studies, parental divorce has been shown to foster chronic anxiety in children, characterized by persistent uncertainty and future-oriented fears (47).

In addition, we analyzed several significant risk factors associated with NSSI, with gender emerging as a particularly noteworthy correlate. Compared to boys, girls are more likely to engage in NSSI behaviors. However, the nature and magnitude of gender differences in NSSI prevalence remain a subject of ongoing academic debate. While some studies have consistently reported higher NSSI prevalence rates among female adolescents (58, 59), other investigations have found minimal or nonsignificant gender differences (60). Notably, a comprehensive meta-analysis focusing on Chinese middle school populations revealed an unexpected pattern, with boys exhibiting higher NSSI detection rates than girls (61). These inconsistent findings across studies may be attributable to methodological variations in assessment tools, sampling strategies, and cultural factors.

Moreover, our findings indicate that the average age of NSSI onset was 15 years, consistent with previous reports (62). This study further confirms the relationship between age and NSSI: the incidence of NSSI tends to increase with age, which is consistent with previous studies (24). However, some researchers suggest that the prevalence of NSSI gradually declines from adolescence into early adulthood (63). The varying results may be due to differences in age groups or the lack of longitudinal data.

Therefore, our study contributes to the literature by demonstrating that in the context of Rural Western China, the association between parental divorce and NSSI remains significant even when accounting for emotional symptoms, pointing to the necessity of exploring these alternative, more direct pathways in future research. Clinically, this suggests that interventions for adolescents from divorced families should not only focus on emotional symptom reduction but also directly address issues like family conflict and improve parenting competencies.

### Strengths and limitations

This study exhibits several notable strengths. The primary methodological strength lies in the application of the PSM approach to control for potential confounding factors, including gender, age, living environment, and parental educational qualification. This approach allows for the effective matching of individuals with similar demographic characteristics. The study’s design, which incorporates class-level analysis complemented by PSM, represents a robust methodological framework that significantly enhances the accuracy and reliability of the research outcomes. Furthermore, the application of PSM in investigating NSSI among adolescents constitutes a novel methodological contribution, as this approach remains relatively underutilized in this specific research domain.

However, the study also has some limitations. First, the cross-sectional nature of the study design inherently limits the ability to establish causal relationships between variables. Second, the reliance on self-reported measures for assessing self-harm behaviors may introduce validity concerns, particularly regarding potential social desirability bias that could affect participants’ willingness to disclose sensitive information. Nevertheless, the school-based survey setting likely mitigates this bias to some extent. Third, the study’s sample size limitations precluded the ability to differentiate between non-divorced parents who have lost a spouse, although this limitation is unlikely to substantially impact the overall findings.

Additionally, the reliability of PSM is dependent on the observed covariates, and observational studies may suffer from omitted variables, leading to potential selection bias. Therefore, it is crucial to include as many relevant factors as possible in the demographic data to reduce selection bias in PSM. Despite these limitations, the study’s large sample size is a significant strength, which enhances the generalizability of the findings.

In conclusion, our findings demonstrate a significant association between parental divorce and increased risk of NSSI among Chinese adolescents, with depression, anxiety, and stress serving as important factors. These results have several important implications for both clinical practice and future research. First, they highlight the need for routine screening for psychological distress and NSSI behaviors among adolescents from divorced families in clinical settings. Second, they suggest that interventions targeting emotion regulation and coping skills may be particularly beneficial for this vulnerable population. Finally, our study underscores the importance of developing family-centered support programs that address the unique challenges faced by divorced families in China. Future longitudinal studies are needed to confirm these associations and explore potential cultural factors influencing the relationship between family structure and adolescent mental health outcomes.

## Conclusion

In summary, our findings indicate that Divorced family, depression, anxiety, and stress each serve as significant and independent risk factors for NSSI among Chinese adolescents. Although no mediating effect through emotional pathways was established, depression, anxiety, and stress remain strong independent predictors of NSSI, underscoring their crucial role in prevention and intervention efforts. Importantly, adolescents from divorced families demonstrated a substantially higher risk of engaging in NSSI compared to their peers from intact families. While this association warrants further validation through longitudinal studies, our results robustly support the importance of familial structural factors in adolescent mental health. These findings highlight the necessity of incorporating targeted support systems—such as school-based psychological services and community-based programs—specifically designed for adolescents experiencing family disruptions.

## Data Availability

The raw data supporting the conclusions of this article will be made available by the authors, without undue reservation.
